# A multi-centre, retrospective case series of oocyte cryopreservation in unmarried women diagnosed with haematological malignancies

**DOI:** 10.1093/hropen/hoaa064

**Published:** 2021-01-16

**Authors:** K Kato, M Ochi, Y Nakamura, H Kamiya, T Utsunomiya, K Yano, Y Michikura, T Hara, K Kyono, K Takeuchi, T Nakayama, J Iwamasa, Y Mio, T Kuramoto, Y Nagata, T Jo, Y Asada, H Ohishi, H Osada, H Yoshida

**Affiliations:** 1 Kato Ladies Clinic, Tokyo 160-0023, Japan; 2 Japan Association of Private Assisted Reproductive Technology Clinics and Laboratories (Japan A-PART), Tokyo 160-0023, Japan; 3 Ochi Yume Clinic Nagoya, Nagoya, Aichi 460-0002, Japan; 4 Nakamura Ladies Clinic, Suita, Osaka 564-0051, Japan; 5 Kamiya Ladies Clinic, Sapporo, Hokkaido 060-0003, Japan; 6 St. Luke Clinic, Oita, 870-0823 Japan; 7 Yano Maternity Clinic, Matsuyama, Ehime 790-0872, Japan; 8 Kanazawa Tamago Clinic, Kanazawa, Ishikawa 920-0016, Japan; 9 Hiroshima Prefectural Hospital, Hiroshima 734-8530, Japan; 10 Kyono ART Clinic Sendai, Sendai, Miyagi 980-0014, Japan; 11 Takeuchi Ladies Clinic, Aira, Kagoshima 899-5421, Japan; 12 Adachi Hospital, Chuo-ku, Kyoto 604-0837, Japan; 13 Sofia Ladies Clinic Suidocho, Chuo-ku, Kumamoto 860-0844, Japan; 14 Mio Fertility Clinic, Yonago, Totttori 683-0008, Japan; 15 Kuramoto Women’s Clinic, Hakata-ku, Fukuoka 812-0013, Japan; 16 IVF Nagata Clinic, Chuo-ku, Fukuoka 810-0001, Japan; 17 Jo Clinic, Nishinomiya, Hyogo 860-0844, Japan; 18 Asada Ladies Clinic, Nagoya, Aichi 450-0002, Japan; 19 Hamanomachi Hospital, Fukuoka 810-0072, Japan; 20 Natural ART Clinic Nihombashi, Chuo-ku, Tokyo 103-6008, Japan; 21 Sendai ART Clinic, Sendai, Miyagi 983-0864, Japan

**Keywords:** haematological malignancies, oocyte cryopreservation, vitrification, post-pubertal women, fertility preservation

## Abstract

**STUDY QUESTION:**

Is oocyte cryopreservation an applicable option for fertility preservation in unmarried patients with haematological malignancies?

**SUMMARY ANSWER:**

Oocyte cryopreservation via the vitrification method is accessible and may be considered an option for fertility preservation in unmarried patients with haematological malignancies.

**WHAT IS KNOWN ALREADY:**

Haematological malignancies are most commonly observed amongst adolescent and young adult women. Although the survival rate and life expectancy of those with haematological malignancies have improved, chemotherapy and radiotherapy may impair their reproductive potential. Oocyte cryopreservation is thus an ideal option to preserve their fertility.

**STUDY DESIGN, SIZE, DURATION:**

This study retrospectively evaluated 193 unmarried patients (age: 26.2 ± 0.4 years) with haematological malignancies, who consulted for oocyte cryopreservation across 20 different fertility centres in Japan between February 2007 and January 2015. The primary outcome measures were the oocyte retrievals and oocyte cryopreservation outcomes. The secondary outcome measures were the outcomes following oocyte warming for IVF.

**PARTICIPANTS/MATERIALS, SETTING, METHODS:**

The patients had commenced ovarian stimulation cycles via antagonist, agonist, natural and minimal methods for oocyte retrievals, defined according to the treatment strategy of each respective fertility centre. A vitrification method using the Cryotop safety kit was used for oocyte cryopreservation. ICSIs were used for insemination of warmed oocytes. The endometrial preparation method for embryo transfer was hormonal replacement therapy, except in the case of a patient who underwent a spontaneous ovulatory cycle.

**MAIN RESULTS AND THE ROLE OF CHANCE:**

Among 193 patients, acute myeloid leukaemia (n = 45, 23.3%) was most common, followed by acute lymphoid leukaemia (n = 38, 19.7%) and Hodgkin’s lymphoma (n = 30, 15.5%). In total, 162 patients (83.9%) underwent oocyte retrieval, and oocytes were successfully cryopreserved for 155 patients (80.3%). The mean number of oocyte retrieval cycles and cryopreserved oocytes were 1.7 ± 0.2 and 6.3 ± 0.4, respectively. As of December 2019, 14 patients (9.2%) had requested oocyte warming for IVF. The survival rate of oocytes after vitrification-warming was 85.2% (75/88). The rates of fertilisation and embryo development were 80.0% (60/75) and 46.7% (28/60), respectively. Ten patients (71.4%) had successful embryo transfers, and seven live births (50.0%) were achieved.

**LIMITATIONS, REASONS FOR CAUTION:**

This study was limited by its retrospective nature. Additionally, there remains an insufficient number of cases regarding the warming of vitrified oocytes to reliably conclude whether oocyte cryopreservation is effective for patients with haematological malignancies. Further long-term follow-up study is required.

**WIDER IMPLICATIONS OF THE FINDINGS:**

Oocyte retrieval and oocyte cryopreservation were accessible for patients with haematological malignancies; however, the number of oocyte retrievals may have been limited due to the initiation of cancer treatments. Acceptable embryonic and pregnancy outcomes could be achieved following oocyte warming; therefore, our results suggest that oocyte cryopreservation can be considered an option for fertility preservation in patients with haematological malignancies.

**STUDY FUNDING/COMPETING INTERESTS:**

This research received no specific grant from any funding agency in the public, commercial or not-for-profit sectors. The authors declare no conflict of interest.

**TRIAL REGISTRATION NUMBER:**

N/A

WHAT DOES THIS MEAN FOR PATIENTS?

Haematological malignancies are most commonly observed amongst adolescent and young adult women. With advancements in diagnostic and treatment methods for those with haematological malignancies, survival rates and life expectancy have improved. Contrary to these improvements, patients remain vulnerable to the declining reproductive potential associated with chemotherapy and radiotherapy. Fertility preservations, such as oocyte, embryo and ovarian tissue cryopreservation, are ideal options for supporting the survivor’s quality of life.

In this case series, the outcomes of oocyte cryopreservation via vitrification and the usage rate of these cryopreserved oocytes by unmarried women with haematological malignancies were reported. Oocyte cryopreservation is achievable for those with haematological malignancies; however, oocyte retrieval cycles were sometimes limited due to initiation of cancer treatments. Additionally, acceptable embryonic and pregnancy outcomes can be achieved following the warming of oocytes. Therefore, these findings provide useful information for oncologists, fertility specialists and clinical psychologists for consultations with patients regarding fertility preservation options.

## Introduction

Haematological malignancies are most common amongst adolescent and young adult (AYA) women in Japan (Katanoda *et al.*, 2017). Due to improvements in diagnostic and treatment methods for those with haematological malignancies, survival rates and life expectancies have dramatically improved. Nonetheless, an increasing number of patients of reproductive age, especially AYAs, are referred for fertility preservation. Fertility preservation options for women include cryopreservation of oocytes (Demirtas *et al.*, 2008; Grifo and Noyes, 2010; Maman *et al.*, 2011; Loren *et al.*, 2013; Schüring *et al.*, 2018; von Wolff *et al.*, 2018), embryos (Herrero *et al.*, 2011; Loren *et al.*, 2013; Konc *et al.*, 2014; Schüring *et al.*, 2018; von Wolff *et al.*, 2018) or ovarian tissue (Donnez *et al.*, 2004; Loren *et al.*, 2013; Suzuki *et al.*, 2015; Schüring *et al.*, 2018; von Wolff *et al.*, 2018). Ovarian tissue cryopreservation is the main option available for patients who cannot delay cancer treatment, as well as pre-pubertal patients. In women with haematological malignancies, however, the preservation of ovarian tissue may reintroduce residual malignant cells (Dolmans *et al.*, 2010; Dolmans *et al.*, 2013; Peek *et al.*, 2015; von Wolff *et al.*, 2018). This may lead to a recurrence upon transplantation, although it has been suggested that the risk of recurrence is low in patients with Hodgkin’s lymphoma (Dolmans *et al.*, 2013; von Wolff *et al.*, 2018). Oocyte and embryo cryopreservation are therefore the currently favoured options for fertility preservation in these patients, with oocyte cryopreservation being the only option for those without a partner, a scenario often observed amongst AYA women.

In July 2005, we raised an issue to the Japan Society of Obstetrics and Gynecology (JSOG) concerning the importance of oocyte cryopreservation in preserving the fertility of unmarried women with haematological malignancies. At the time, the clinical application of ART for these patients was not recommended in Japan by the JSOG as they believed that ART should only be available to married women who were physically and mentally capable of carrying and delivering babies. This remained until the American Society for Reproductive Medicine (ASRM) announced that oocyte cryopreservation was in its ‘experimental phase’ (The Practice Committee of the American Society for Reproductive Medicine and Practice Committee of the Society for Assisted Reproductive Technology, 2008). As a result, the JSOG allowed research (January 2007) following the premise that oocyte cryopreservation could be an option for preserving the fertility of unmarried women suffering from haematological malignancies.

We have studied oocyte cryopreservation performed for unmarried women with haematopoietic malignancies between February 2007 and January 2015. First, the outcomes of oocyte cryopreservation amongst the cohort were recorded; this was followed by follow-up inquiries regarding pregnancy outcomes as well as oocyte storage status.

## Materials and methods

These procedures took place between February 2007 and January 2015; 193 unmarried patients (age: 26.2 ± 0.4 years old) with haematopoietic malignancies consulted at 20 different fertility centres. The study was approved by the JSOG’s Institutional Review Board, with written informed consent obtained from all patients.

## Results

**Figure 1.**
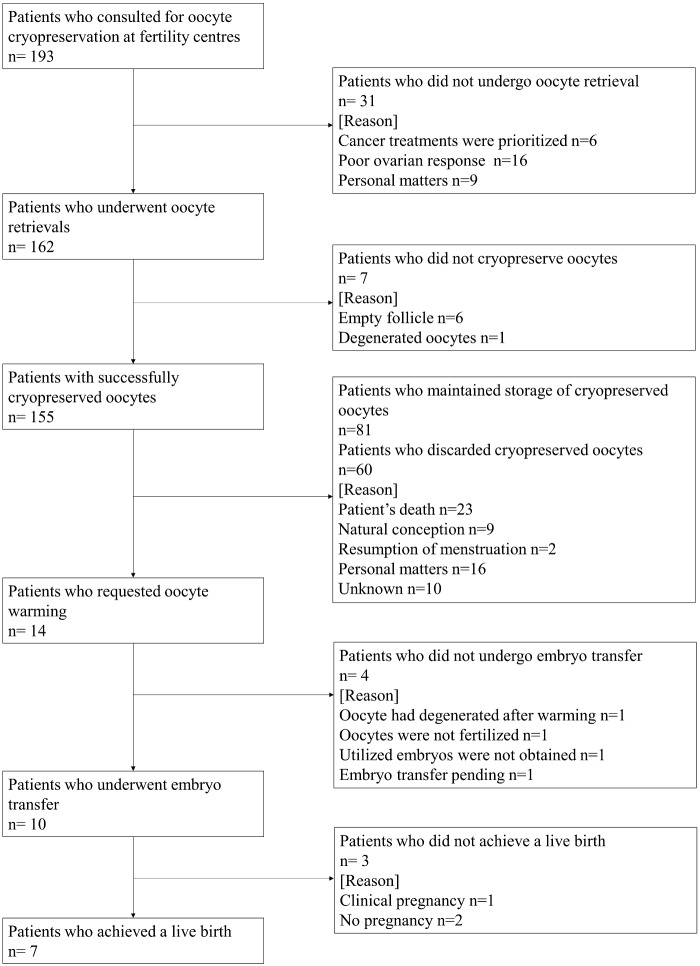
Flow chart of all patients.

Although the 20 fertility centres were located across Japan, cases of oocyte cryopreservation were most prevalent in urban areas (Tokyo: 74 patients, Nagoya: 21 patients and Osaka: 18 patients; data not shown). Patient characteristics, diagnosis and outcomes of oocyte cryopreservation are shown in Table I. Acute myeloid leukaemia (AML, n = 45, 23.3%) was most common, followed by acute lymphoid leukaemia (ALL, n = 38, 19.7%) and Hodgkin’s lymphoma (HL, n = 30, 15.5%, Table I). A flow chart of all patients and their destinations are shown in Fig. 1. Of the 193 patients, 94 patients had previously undergone at least one course of cancer treatment to maintain remission (Supplementary Table SI). Ultrasound-guided transvaginal oocyte retrievals (oocyte retrievals) were performed on 162 patients (83.9%) who had commenced ovarian stimulation cycles via antagonist, agonist, natural and minimal methods, defined according to the treatment strategy of each respective fertility centre; the mean number of OR cycles per patients was 1.7 ± 0.2. Among all subjects, 155 patients (80.3%) achieved successful cryopreservation of their oocytes; the mean number of cryopreserved oocytes per patient was 6.3 ± 0.4 (Table I). Supplementary Table SII shows the outcomes of the oocyte retrievals according to the ovarian stimulation method. There were 97 patients (59.9%) who underwent minimal stimulation cycles; the mean number of cycles and cryopreserved oocytes were 1.4 ± 0.1 and 5.0 ± 0.5, respectively. Vitrification using Cryotop Safety Kits (Kitazato corporation) were used for oocyte cryopreservation (Kato *et al.*, 2012).

**Table I:** Diagnosis of patients and the oocyte cryopreservation outcomes.

Diagnosis^*^	No. of patients consulted	(%)	Age (mean ± SE, min-max)	No. of patients for whom oocyte retrieval (OR) cycle was started	(%)	No. of OR cycles per patient (mean ± SE)	No. of patients with cryopreserved oocytes	(%)	No. of oocytes cryopreserved per patient (mean ± SE)
AML	45	23.3	26.1 ± 0.8 (16–40)	32	71.1	1.8 ± 0.2	31	68.9	6.0 ± 0.8
ALL	38	19.7	25.3 ± 0.9 (17–37)	29	76.3	1.5 ± 0.2	28	73.7	6.0 ± 1.1
HL	30	15.5	26.1 ± 0.8 (19–36)	29	96.7	1.2 ± 0.1	29	96.7	6.3 ± 1.0
MDS	20	10.4	26.9 ± 1.2 (17–37)	19	95.0	1.7 ± 0.2	17	85.0	7.1 ± 1.6
NHL	17	8.8	27.9 ± 1.3 (18–35)	13	76.5	1.4 ± 0.3	11	64.7	4.5 ± 1.0
ML	12	6.2	26.6 ± 1.9 (19–39)	12	100	2.0 ± 0.8	11	91.7	6.7 ± 2.4
AA	11	5.7	25.7 ± 1.7 (19–35)	11	100	2.9 ± 1.6	11	100	6.3 ± 1.9
CML	4	2.1	21.9 ± 1.7 (18–26)	3	75.0	1.3 ± 0.3	3	75.0	9.3 ± 3.7
MM	3	1.6	36.6 ± 0.4 (36–37)	2	66.7	1.5 ± 0.5	2	66.7	10.5 ± 2.5
APL	2	1.0	22.6 ± 1.7 (21, 24)	2	100	1.5 ± 0.5	2	100	3.5 ± 0.5
B-LBL	2	1.0	23.8 ± 0.8 (23, 25)	2	100	1.0	2	100	9.5 ± 1.5
Ph+ALL	2	1.0	22.8 ± 1.2 (22, 24)	1	50.0	1.0	1	50.0	1.0
T-LBL	2	1.0	33.4 ± 1.8 (32, 35)	2	100	6.0 ± 5.0	2	100	5.5 ± 4.5
Others	5	2.6	24.4 ± 2.2 (18–32)	5	100	1.2 ± 0.2	5	100	8.4 ± 1.6
Total	193	–	26.2 ± 0.4 (16–40)	162	83.9	1.7 ± 0.2	155	80.3	6.3 ± 0.4

* AML, acute myeloid leukaemia; ALL, acute lymphoid leukaemia; HL, Hodgkin’s lymphoma; MDS, myelodysplastic syndromes; NHL, non-Hodgkin’s lymphoma; ML, malignant lymphoma (type unknown); AA, aplastic anaemia; CML, chronic myeloid leukaemia; MM, multiple myeloma; APL, acute promyelocytic leukaemia; B-LBL, B-lymphoblastic lymphoma; Ph + ALL, Philadelphia chromosome-positive acute lymphoblastic leukaemia; T-LBL, T-cell lymphoblastic lymphoma; Others: chronic active Epstein-Barr virus (CAEBV) infection, chronic granulomatous disease (CGD), essential thrombocythemia (ET) and primary myelofibrosis.

As of December 2019, 14 patients (9.2%, 14/152) were considered free of disease and requested oocyte warming. The patients’ diagnoses were CML (n = 1), NHL (n = 1), AA (n = 1), ALL (n = 5), AML (n = 4), T-LBL (n = 1) and MDS (n = 1, Table II). The mean age of the 14 patients at oocyte vitrification and oocyte warming was 29.5 ± 1.1 and 34.4 ± 1.2 years, respectively. Excluding cases 3 and 14, these patients underwent at least one course of cancer treatment prior to visiting the fertility centres; the average duration of storage was 65.5 ± 5.0 months. Oocyte warming was performed at the same centre where OR and vitrification had taken place. A total of 88 oocytes were warmed, with 75 oocytes having survived (85.2%). ICSIs were used for insemination; the semen analysis data of the partners are shown in Supplementary Table SIII. The rates of fertilisation and embryos development were 80.0% (60/75) and 46.7% (28/60), respectively (Table II). Ten patients (71.4%) underwent a total of 17 single embryo transfer cycles including seven fresh cleavage-stage embryo transfer cycles, two vitrified-warmed cleavage-stage embryo transfer cycles and eight vitrified-warmed blastocyst transfer cycles. The endometrial preparation method for embryo transfer was hormonal replacement therapy, except in the case of one patient who underwent a spontaneous ovulatory cycle due to resumption of menstruation (case 2). A total of seven live births occurred, absent of any obvious malformation. The clinical pregnancies, determined by the gestational sac observation at 6–7 weeks, and the live births after 22 weeks of pregnancy, were divided by the embryo transfer cycles, resulting in clinical pregnancy and live birth rates of 52.9% (9/17) and 41.2% (7/17), respectively (Table II).

**Table II:** Clinical outcomes following oocyte vitrification-warming and embryo transfer in women with haematopoietic malignancies.

Case	Type of malignancy^*^	Woman's age at oocyte cryopreservation	No. of oocytes vitrified	Woman's age at oocyte warming	Duration of stage (months)	No. of oocytes warmed	No. of survived oocytes	(%)	No. of oocytes fertilised	(%)	No. of utilised embryos developed (embryonic stage)	(%)	No. of embryo transfer cycles	No. of embryos transferred per cycle	Embryonic stage at transfer	Clinical pregnancy (gestational sac observation)	(%)	Live birth	(%)	Comment
1	CML	23	13	30	87	13	9	69.2	9	100.0	4 (2: 5-cell, 1: 7-cell, 1: blastocyst)	44.4	1	1	7-cell (fresh)	0	0	–	–	3 surplus embryos were vitrified
2	NHL	30	7	34	53	7	6	85.7	6	100.0	4 (3:4-cell, 1:blastocyst)	66.7	4	1	4-cell (fresh)	1	50	0	25	–
														1	Blastocyst (vitrified-warmed)	1		1		–
														1	4-cell ( vitrified-warmed)	0		–		–
														1	4-cell (vitrified-warmed)	0		–		–
3	AA	28	3	36	103	3	3	100.0	3	100.0	2(1: 8-cell, 1: blastocyst)	66.7	1	1	8-cell (fresh)	1	100	1	100	1 surplus embryos were vitrified
4	ALL	36	3	41	67	3	1	33.3	1	100.0	0	0	–	–	–	–	–	–	–	–
5	ALL	30	9	35	62	9	8	88.9	7	87.5	2(1: 8-cell, 1: blastocyst)	28.6	1	1	8-cell (fresh)	1	100	1	100	1 surplus embryos were vitrified
6	AML	30	7	34	52	7	7	100.0	7	100.0	2(1: 8-cell, 1: blastocyst)	28.6	2	1	8-cell (fresh)	0	50	–	50	–
														1	Blastocyst (vitrified-warmed)	1		1		–
7	AML	30	8	34	59	8	8	100.0	8	100.0	3(3: blastocysts)	37.5	2	1	Blastocyst (vitrified-warmed)	0	50	–	50	–
														1	Blastocyst (vitrified-warmed)	1		1		–
8	AML	27	8	31	61	8	8	100.0	7	87.5	3(3: blastocysts)	42.9	3	1	Blastocyst (vitrified-warmed)	1	33.3	0	0	–
														1	Blastocyst (vitrified-warmed)	0		–		–
														1	Blastocyst (vitrified-warmed)	0		–		–
9	ALL	35	10	41	80	10	9	90.0	3	33.3	3(3: blastocysts)	100	1	1	Blastocyst (vitrified-warmed)	1	100	1	100	1 surplus embryos were vitrified
10	T-LBL	35	10	42	87	4	4	100.0	0	0.0	–	–	–	–	–	–	–	–	–	6 oocytes were still stored
11	AML	31	1	36	65	1	0	0.0	–	–	–	–	–	–	–	–	–	–	–	–
12	ALL	31	4	33	28	4	3	75.0	3	100.0	3(1:8-cell, 2: blastocysts)	100	–	–	–	–	–	–	–	Embryo transfer was not performed
13	ALL	23	7	27	54	4	4	100.0	3	75.0	1(5-cell)	33.3	1	1	5-cell (fresh)	0	0	–	–	3 oocytes were still stored
14	MDS	24	22	28	56	7	5	71.4	3	60.0	1 (8-cell)	33.3	1	1	8-cell (fresh)	1	100	1	100	15 oocytes were still stored
Total	–	29.5 ± 1.1	112	34.4 ± 1.2	65.5 ± 5.0	88	75	85.2	60	80.0	28	46.7	17	17	–	9	52.9	7	41.2	–

* CML, chronic myeloid leukaemia; NHL, non-Hodgkin’s lymphoma; AA, aplastic anaemia; ALL, acute lymphoid leukaemia; AML, acute myeloid leukaemia; T-LBL, T-cell lymphoblastic lymphoma; MDS, myelodysplastic syndromes.

Among the 155 patients, 81 (52.2%) are currently maintaining storage of their cryopreserved oocytes; the mean patient age and duration of storage as of December 2019 was 34.5 ± 0.6 years and 98.2 ± 3.5 months, respectively (data not shown). A total of 60 patients (38.7%) requested that their cryopreserved oocytes be discarded, either due to the patient’s death (n = 23, 38.3%), personal matters (n = 16, 26.7%), natural conception (n = 9, 15.0%), resumption of menstruation (n = 2, 3.3%) or unknown reasons (n = 10, 16.7%, Fig. 1).

## Discussion

According to the Japanese epidemiology study published in 2017 (Katanoda *et al.*, 2017), the incidence of cancer in the AYA generation (age, 15–39 years) in Japan 2011 was 20 258, which included 6868 male (33.9%) and 13 390 female (66.1%) individuals. This suggests that the incidence of cancer is more frequent in women in the AYA generation. Cancer chemotherapy and radiotherapy both negatively affect reproductive potential, although the degree to which this has an effect varies depending on the type of drug, dose and patient age (Lee *et al.*, 2006; Meirow *et al.*, 2010). These adverse effects are usually irreversible (Bar-Joseph *et al.*, 2010; Ezoe *et al.*, 2014); therefore, oocyte or embryo cryopreservation is the preferable option to preserve fertility. The most frequently occurring cancers in Japan are breast cancer, in women aged 30–39 years, and haematopoietic malignancies in women aged 15–29 years (Katanoda *et al.*, 2017). With breast cancer, patients can usually can wait between 4 and 6 weeks between surgery and chemotherapy, which provides an acceptable amount of time to perform oocyte retrievals (Shapira *et al.*, 2015). By contrast, haematopoietic malignancies often require early treatment initiation; therefore, patients with haematopoietic malignancies have a more limited timeframe for oocyte retrievals. In the present study, among the 193 patients who consulted in fertility centres across Japan, 94 patients had already received a form of cancer treatment prior to visiting the fertility centre; 22 of these patients (23.4%) could not undergo oocyte retrievals. By contrast, among the 97 patients who had not received prior cancer treatment, only eight patients had failed oocyte retrievals (8.2%, Supplementary Table SI). This suggests the possibility of ovarian insufficiency associated with cancer treatment and supports the efficient initiation of oocyte cryopreservation in the limited timeframes. Taking into account that the mean number of oocyte retrieval cycles per patient in the present cohort was 1.7 ± 0.2, the maximum number of oocyte retrievals could therefore be limited to two cycles in the patients with haematopoietic malignancies.

The long-term follow-up of cancer patient outcomes after embryo transfer using cryopreserved oocytes has rarely been documented due to the limited number of patients who are both in remission and seeking to be pregnant. As of December 2019, 14 patients had requested oocytes to be warmed for IVF, with a cryopreserved oocyte usage rate of 9.0%. Garcia-Velasco *et al.* (2013) and Martinez *et al.* (2014) reported the use of cryopreserved oocytes by cancer patients as 1.1% (4/355) and 3.1% (11/357), respectively, whereas the usage rate we report is higher (9.0%). The reason behind these differences is unknown; however, we suspect follow-up duration to be a cause. The live birth rate per embryo transfer and per warmed oocyte was 41.2% (7/17) and 8.3% (7/84), respectively, suggesting an optimistic live birth rate for haematopoietic malignancy patients. Currently, 81 patients have preserved oocytes. Considering their average age (34.5 ± 0.6 years old), warming oocytes for IVF remains a possible option for these patients; therefore, further long-term follow-up study is required.

In conclusion, the present study indicates that oocyte cryopreservation via vitrification for patients with haematopoietic malignancies is achievable; however, due to the limited amount of time between diagnosis and treatment, the number of retrieved oocytes could be limited. When oocytes are successfully retrieved, embryonic and pregnancy outcomes of the vitrified oocytes in these patients are acceptable. Although there is still an insufficient number of oocyte warming cases to be conclusive, and further long-term follow-up is required, our results suggest that oocyte cryopreservation can be considered an option for fertility preservation for patients with haematological malignancies. Our study offers time-sensitive guidance for oncologists, fertility specialists and clinical psychologists in weighing fertility preservation options with their patients.

## Supplementary data

Supplementary data are available at *Human Reproduction Open* online.

## Data availability

The data underlying this article cannot be shared publicly due to the privacy of individuals who participated in the study. The data will be shared on reasonable request to the corresponding author.

## Supplementary Material

hoaa064_Supplementary_Data

## References

[hoaa064-B1] Bar-Joseph H, Ben-Aharon I, Rizel S, Stemmer SM, Tzabari M, Shalgi R Doxorubicin-induced apoptosis in germinal vesicle (GV) oocytes. Reprod Toxicol 2010;30:566–572. 20656019 10.1016/j.reprotox.2010.07.003

[hoaa064-B2] Demirtas E, Elizur SE, Holzer H, Gidoni Y, Son WY, Chian RC, Tan SL Immature oocyte retrieval in the luteal phase to preserve fertility in cancer patients. Reprod Biomed Online 2008;17:520–523. 18854106 10.1016/s1472-6483(10)60239-8

[hoaa064-B3] Dolmans MM, Luyckx V, Donnez J, Andersen CY, Greve T Risk of transferring malignant cells with transplanted frozen-thawed ovarian tissue. Fertil Steril 2013;99:1514–1522. 23541406 10.1016/j.fertnstert.2013.03.027

[hoaa064-B4] Dolmans MM, Marinescu C, Saussoy P, Van Langendonckt A, Amorim C, Donnez J Reimplantation of cryopreserved ovarian tissue from patients with acute lymphoblastic leukemia is potentially unsafe. Blood 2010;116:2908–2914. 20595517 10.1182/blood-2010-01-265751

[hoaa064-B5] Donnez J, Dolmans MM, Demylle D, Jadoul P, Pirard C, Squifflet J, Martinez-Madrid B, van Langendonckt A Livebirth after orthotopic transplantation of cryopreserved ovarian tissue. Lancet 2004;364:1405–1410. 15488215 10.1016/S0140-6736(04)17222-X

[hoaa064-B6] Ezoe K, Murata N, Yabuuchi A, Okuno T, Kobayashi T, Kato O, Kato K Long-term adverse effects of cyclophosphamide on follicular growth and angiogenesis in mouse ovaries. Reprod Biol 2014;14:238–242. 25152523 10.1016/j.repbio.2014.04.007

[hoaa064-B7] Garcia-Velasco JA, Domingo J, Cobo A, Martinez M, Carmona L, Pellicer A Five years' experience using oocyte vitrification to preserve fertility for medical and nonmedical indications. Fertil Steril 2013;99:1994–1999. 23465707 10.1016/j.fertnstert.2013.02.004

[hoaa064-B8] Grifo JA, Noyes N Delivery rate using cryopreserved oocytes is comparable to conventional in vitro fertilization using fresh oocytes: potential fertility preservation for female cancer patients. Fertil Steril 2010;93:391–396. 19439285 10.1016/j.fertnstert.2009.02.067

[hoaa064-B9] Herrero L, Martinez M, Garcia-Velasco JA Current status of human oocyte and embryo cryopreservation. Curr Opin Obstet Gynecol 2011;23:245–250. 21734500 10.1097/GCO.0b013e32834874e2

[hoaa064-B10] Katanoda K, Shibata A, Matsuda T, Hori M, Nakata K, Narita Y, Ogawa C, Munakata W, Kawai A, Nishimoto H Childhood, adolescent and young adult cancer incidence in Japan in 2009-2011. Jpn J Clin Oncol 2017;47:762–771. 28541571 10.1093/jjco/hyx070 PMC5896699

[hoaa064-B11] Kato K, Takehara Y, Segawa T, Kawachiya S, Okuno T, Kobayashi T, Bodri D, Kato O Minimal ovarian stimulation combined with elective single embryo transfer policy: age-specific results of a large, single-centre, Japanese cohort. Reprod Biol Endocrinol 2012;10:35. 22541043 10.1186/1477-7827-10-35 PMC3407520

[hoaa064-B12] Konc J, Kanyó K, Kriston R, Somoskői B, Cseh S Cryopreservation of embryos and oocytes in human assisted reproduction. Biomed Res Int 2014;2014:1–9. 10.1155/2014/307268 PMC3980916 24779007

[hoaa064-B13] Lee SJ, Schover LR, Partridge AH, Patrizio P, Wallace WH, Hagerty K, Beck LN, Brennan LV, Oktay K American Society of Clinical Oncology recommendations on fertility preservation in cancer patients. J Clin Oncol 2006;24:2917–2931. 16651642 10.1200/JCO.2006.06.5888

[hoaa064-B14] Loren AW, Mangu PB, Beck LN, Brennan L, Magdalinski AJ, Partridge AH, Quinn G, Wallace WH, Oktay K, American Society of Clinical Oncology. Fertility preservation for patients with cancer: American Society. J Clin Oncol 2013;31:2500–2510. 23715580 10.1200/JCO.2013.49.2678 PMC5321083

[hoaa064-B15] Maman E, Meirow D, Brengauz M, Raanani H, Dor J, Hourvitz A Luteal phase oocyte retrieval and in vitro maturation is an optional procedure for urgent fertility preservation. Fertil Steril 2011;95:64–67. 20688325 10.1016/j.fertnstert.2010.06.064

[hoaa064-B16] Martinez M, Rabadan S, Domingo J, Cobo A, Pellicer A, Garcia-Velasco JA Obstetric outcome after oocyte vitrification and warming for fertility preservation in women with cancer. Reprod Biomed Online 2014;29:722–728. 25444506 10.1016/j.rbmo.2014.09.002

[hoaa064-B17] Meirow D, Biederman H, Anderson RA, Wallace WH Toxicity of chemotherapy and radiation on female reproduction. Clin Obstet Gynecol 2010;53:727–739. 21048440 10.1097/GRF.0b013e3181f96b54

[hoaa064-B18] Peek R, Bastings L, Westphal JR, Massuger LF, Braat DD, Beerendonk CC A preliminary study on a new model system to evaluate tumour-detection and tumour-purging protocols in ovarian cortex tissue intended for fertility preservation. Hum Reprod 2015;30:870–876. 25662807 10.1093/humrep/dev013

[hoaa064-B19] Schüring AN, Fehm T, Behringer K, Goeckenjan M, Wimberger P, Henes M, Henes J, Fey MF, von Wolff M Practical recommendations for fertility preservation in women by the FertiPROTEKT network. Part I: indications for fertility preservation. Arch Gynecol Obstet 2018;297:241–255. 29177593 10.1007/s00404-017-4594-3 PMC5762797

[hoaa064-B20] Shapira M, Raanani H, Meirow D IVF for fertility preservation in breast cancer patients–efficacy and safety issues. J Assist Reprod Genet 2015;32:1171–1178. 26126877 10.1007/s10815-015-0519-x PMC4554381

[hoaa064-B21] Suzuki N, Yoshioka N, Takae S, Sugishita Y, Tamura M, Hashimoto S, Morimoto Y, Kawamura K Successful fertility preservation following ovarian tissue vitrification in patients with primary ovarian insufficiency. Hum Reprod 2015;30:608–615. 25567618 10.1093/humrep/deu353

[hoaa064-B22] The Practice Committee of the American Society for Reproductive Medicine, Practice Committee of the Society for Assisted Reproductive Technology. Ovarian tissue and oocyte cryopreservation. Fertil Steril 2008;90:S241–S246. 17055811 10.1016/j.fertnstert.2006.08.083

[hoaa064-B23] von Wolff M, Germeyer A, Liebenthron J, Korell M, Nawroth F Practical recommendations for fertility preservation in women by the FertiPROTEKT network. Part II: fertility preservation techniques. Arch Gynecol Obstet 2018;297:257–267. 29181578 10.1007/s00404-017-4595-2 PMC5762782

